# The first complete mitochondrial genome sequence of *Nanorana parkeri* and *Nanorana ventripunctata* (Amphibia: Anura: Dicroglossidae), with related phylogenetic analyses

**DOI:** 10.1002/ece3.4214

**Published:** 2018-06-11

**Authors:** Lichun Jiang, Zhangqiang You, Peng Yu, Qiping Ruan, Wei Chen

**Affiliations:** ^1^ Ecological Security and Protection Key Laboratory of Sichuan Province Mianyang Normal University Mianyang Sichuan China; ^2^ Key Laboratory for Molecular Biology and Biopharmaceutics School of Life Science and Technology Mianyang Normal University Mianyang Sichuan China

**Keywords:** control region, mitochondrial genome, *Nanorana parkeri*, *Nanorana pleskei*, *Nanorana ventripunctata*, phylogenetic analyses

## Abstract

Members of the *Nanorana* genus (family Dicroglossidae) are often referred to as excellent model species with which to study amphibian adaptations to extreme environments and also as excellent keystone taxa for providing insights into the evolution of the Dicroglossidae. However, a complete mitochondrial genome is currently only available for *Nanorana pleskei*. Thus, we analyzed the complete mitochondrial genomes of *Nanorana parkeri* and *Nanorana ventripunctata* to investigate their evolutionary relationships within *Nanorana* and their phylogenetic position in the family Dicroglossidae. Our results showed that the genomes of *N. parkeri* (17,837 bp) and *N. ventripunctata* (18,373 bp) encode 13 protein‐coding genes (PCGs), two ribosomal RNA genes, 23 transfer RNA (tRNA) genes, and a noncoding control region. Overall sequences and genome structure of the two species showed high degree of similarity with *N. pleskei*, although the motif structures and repeat sequences of the putative control region showed clear differences among these three *Nanorana* species. In addition, a tandem repeat of the tRNA‐Met gene was found located between the tRNA‐Gln and ND2 genes. On both the 5′ and 3′‐sides, the control region possessed distinct repeat regions; however, the CSB‐2 motif was not found in *N. pleskei*. Based on the nucleotide sequences of 13 PCGs, our phylogenetic analyses, using Bayesian inference and maximum‐likelihood methods, illustrate the taxonomic status of *Nanorana* with robust support showing that *N. ventripunctata* and *N. pleskei* are more closely related than they are to *N. parkeri*. In conclusion, our analyses provide a more robust and reliable perspective on the evolutionary history of Dicroglossidae than earlier analyses, which used only a single species (*N. pleskei*).

## 1 INTRODUCTION

The *Nanorana* (Amphibia: Anura: Dicroglossidae) are a genus of dicroglossid frogs found over much of Asia including Pakistan, India, Nepal, China, Myanmar, Thailand, Laos, and Vietnam (Frost, 2018). The *Nanorana* genus comprises about 28 species (Frost, 2018), including three species (*Nanorana parkeri*,* Nanorana ventripunctata*, and *Nanorana pleskei*) that are endemic to the Tibetan Plateau in China (Che et al., 2010; Chen, Liu, Jiang, Xie, & Zheng, 2005; Fei, Hu, Ye, & Huang, 2009; Fei, Ye, Huang, Jiang, & Xie, 2005; Fei et al., 2004; Lu & Yang, 2004; Wang, Annemarie, Muhammad, & Xie, 2004). *Nanorana parkeri* is known from southern and eastern Xizang, China, and the Tibetan Plateau of Nepal at elevations of 2,850–5,000 m asl. It has recently been reported from Kashmir in northern India, but the distribution within this region needs further confirmation. *Nanorana ventripunctata* is endemic to northwestern Yunnan province in China, ranging in elevation from 3,120 to 4,100 m asl, while *Nanorana pleskei* is known from Qinghai, Gansu, and Sichuan provinces in China, at elevations of 3,300–4,500 m asl (Fei et al., 2004). Similar to most anurans, *Nanorana* species have a terrestrial adult life history. However, due to their high‐elevation habitats, *Nanorana* species experience extremely harsh abiotic factors, including hypoxia, high UV radiation, and dramatic temperature changes on a daily basis. Consequently, *Nanorana* is an excellent model species for studying the adaptations of frogs to extreme environmental conditions (Sun et al., 2015). Does the unique high‐elevation environment of *Nanorana* have a greater impact on species differentiation and gene sequences characteristics? Our study aimed to clarify the mitochondrial genome sequence characteristics and phylogenetic relationship and the taxonomic status of the three species in the genus *Nanorana*.

The phylogenetic relationships of *Nanorana* have been studied previously (Che et al., 2009; Chen, Wang, Liu, Xie, & Jiang, 2011; Lu, 1995; Zhou et al., 2014); however, debates on the taxonomic status of the three species that are the focus of this study are still ongoing. The taxonomy of *Nanorana* species is not yet fully settled because of numerous changes during the last decade. Previous phylogenetic analyses support *N. pleskei* and *Quasipaa spinosa* as having a close relationship (Chen et al., 2011), as well as *N. ventripunctata* and *N. parkeri* (*N. pleskei *+ (*N. parkeri *+ *N. ventripunctata*)) (Lu, 1995). In other literature, however, *N. pleskei* and *N. ventripunctata* are reported to have a closer relationship (*N. parkeri *+ (*N. pleskei *+ *N. ventripunctata*)) (Che et al., 2009; Zhou et al., 2014), while Pyron and Wiens (2011) thought that *N. pleskei* and *N. parkeri* had a closer evolutionary relationship (*N. ventripunctata *+ (*N. pleskei *+ *N. parkeri*)). Thus, complete sequencing of the mtDNA in *Nanorana* can help clarify the phylogenetic relationships and genetic diversity within the genus. With that information, we can then better understand the phylogenetic status and intraspecific relationships among the three species within this group (Che et al., 2009; Chen et al., 2011; Jiang & Zhou, 2001, 2005; Jiang et al., 2005; Roelants, Jiang, & Bossuyt, 2004).

Mitochondrial genomes have been widely used as molecular markers in phylogenetic and phylogeographic studies of amphibians because of their high mutation and substitution rates, rare gene recombination, maternal transmission pathway, high copy number, and easy accessibility (Bossuyt, Brown, Hillis, Cannatella, & Milinkovitch, 2006; Howlader, Nair, Gopalan, & Merilä, 2015; Jiang et al., 2005; Matsui et al., 2011; de Sá et al., 2012). Moreover, complete mitochondrial genomes are effectively used as molecular markers in studies of population genetics and conservation biology (Ren et al., 2009; Sahoo et al., 2015; San Mauro, Gower, Oommen, Wilkinson, & Zardoya, 2004; Sano, Kurabayashi, Fujii, Yonekawa, & Sumidam, 2004; Yong, Song, Lim, Eamsobhana, & Tan, 2016). For example, complete mitochondrial genomes have been used to elucidate many evolutionary questions regarding amphibians (Liu, Wang, & Bing, 2005; Yuan, Xia, Zheng, & Zeng, 2016; Zhang, Nie, Wang, & Hu, 2009), as well as to investigate the evolutionary relationships of endangered species, such as *Odorrana ishikawae*,* Mantella madagascariensis*,* Andrias davidianus*, and *Paa spinosa* (Kurabayashi et al., 2006, 2010; Zhang, Chen, Liu, Zhou, & Qu, 2003; Zhou, Zhang, Zheng, Yu, & Yang, 2009).

Mitochondrial genes such as the COX I, Cytochrome b (Cytb), D‐loop, tRNA, and NADH have been used for previous phylogenetic and phylogeographic studies on the genetic divergence of *Nanorana* (Che et al., 2010; Liu et al., 2015; Wang et al., 2013; Zhang et al., 2010; Zhou et al., 2014). Here, we use complete mitochondrial genomes to analyze the phylogenetic relationships of the three *Nanorana* species (*N. parkeri*,* N. ventripunctata*, and *N. pleskei*) and other related species. Moreover, in order to reconstruct a robust evolutionary relationship among the three species, we need additional mitochondrial genomic information from *Nanorana* species. Therefore, we sequenced the complete mitochondrial genome of *N. parkeri* and *N. ventripunctata* and summarized the structural variations of 40 mitochondrial genome sequences in the Family Dicroglossidae. We reconstructed the phylogenetic relationships of Dicroglossidae using the concatenated sequences of 13 protein‐coding genes from Dicroglossidae mitochondrial genomes, based on which the evolutionary characteristics of the mitochondrial genomes in Dicroglossidae were evaluated. Furthermore, we analyzed the mitochondrial genomic sequence and phylogenetic relationships within *N. pleskei*,* N. ventripunctata* and *N. parkeri* to assess the evolutionary status of the three species within the *Nanorana* genus. Additionally, the complete mitochondrial genomes of two *Nanorana* species (*N. ventripunctata* and *N. parkeri*) were analyzed to find novel data with which to investigate the placement of the three *Nanorana* species in the phylogenetic tree of Dicroglossidae and to provide molecular data for further study on the taxonomic status and adaptive evolutionary mechanisms of these high‐altitude species.

## 2 MATERIALS AND METHODS

### 2.1 Sampling and DNA extraction

**Figure 1:**
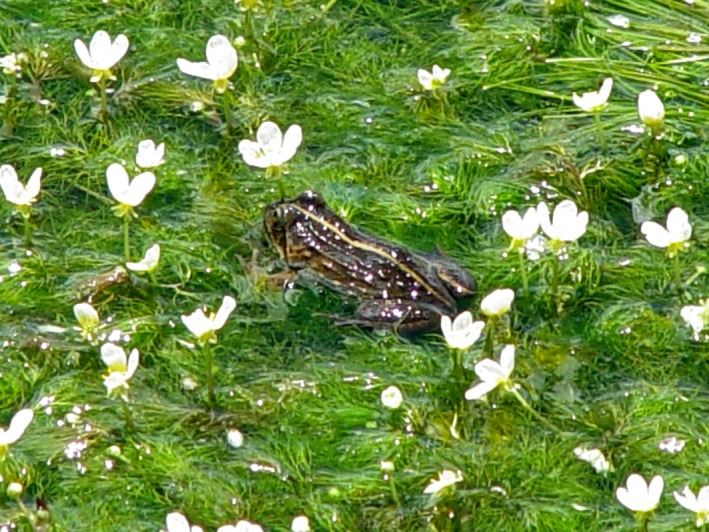
The sample of *Nanorana parkeri* collected in the field. Photographs by XiaoYan Ma

The Xizang Plateau frog (*N. parkeri*, Figure 1) was sampled from Dangxiong County (4,300 m asl), the Tibet Autonomous Region, China, in September 2015. The Yunnan slow frog (*N. ventripunctata*) was sampled from Xianggelila County (4,200 m asl), Yunnan province, China, in July 2016. All collections were initially preserved in 95% ethanol and stored at −70°C until DNA extraction was performed. According to the protocol adopted by Zhang, Chao, Lai, Li, and Zhao (2000) and Xia, Liu, and Lu (2002), total mtDNA of two *Nanorana* species was extracted from skin tissue for the following PCR amplification.

### 2.2 Mitochondrial DNA amplification and sequencing

The entire mitochondrial genome was amplified in twelve overlapping segments by PCR with LA‐Taq DNA Polymerase (TaKaRa, China), using 20 ng of total genomic DNA from the sample as a template. Complete mtDNA was amplified as concatenated sequences by adopting selectively amplified mtDNA templates and 10 primer pairs, as published by Kurabayashi and Sumida (2009). Partial PCR primers were also designed based on the alignments of the relatively conserved regions of congeneric *N. pleskei* (NC_016119) and *N. taihangnica* (NC_024272). The PCR amplification was performed as follows: 2.5 min at 94°C, followed by 30 cycles of 0.5 min at 94°C, 0.5 min at 50–59°C, 3–5 min at 60°C, and a 9 min final extension at 72°C. PCR reactants were loaded on 0.8%–1.0% agarose gels, stained with ethidium bromide and photographed under ultraviolet light. PCR products were purified with Gel Extract Purification Kits (V‐gene) and automated sequencing using an ABI 3730 sequencer, either directly or following subcloning into the pMD18‐T vector (TaKaRa, China). To ensure maximum accuracy, each amplification product was sequenced twice independently, followed by a third PCR amplification.

### 2.3 Sequence assembly and analysis

Sequences were assembled manually and aligned, and each gene was then translated into an amino acid sequence using MEGA 6.0 (Tamura, Stecher, Peterson, Filipski, & Kumar, 2013). The amino acid sequence alignments of each of the protein‐coding genes (PCGs) were generated using the computer program Clustal X 1.83 (Thompson, Gibson, Plewniak, Jeanmougin, & Higgins, 1997). Based on sequence similarity results from BLAST searches, ribosomal RNA (rRNA) genes were recognized, and tRNA genes were identified using tRNAscan‐SE 1.21 (Schattner, Brooks, & Lowe, 2005). Base composition and codon usage were analyzed in MEGA 6.0 (Tamura et al., 2013). The mitochondrial genome sequences have been submitted to NCBI GenBank with the accession number NC_026789 (*N. parkeri*) and KY594708 (*N. ventripunctata*). Features of the base composition of nucleotide sequences were detected using the AT‐skew and GC‐skew in the mitochondrial genome. We then calculated the AT‐skew and GC‐skew using the following formulae from Perna and Kocher (1995): AT‐skew = (A − T)/(A + T) and GC‐skew = (G − C)/(G + C).

### 2.4 Phylogenetic analysis

Combined with 38 other Dicroglossidae mitochondrial genomes from NCBI GenBank (Supporting information Table S1), the mitochondrial genomes of *N. parkeri* and *N. ventripunctata* were analyzed using the phylogenetic tree method, with the concatenated sequences of the 13 protein‐coding genes and the two species *Babina subaspera* (NC_022871) and *Hylarana guentheri* (NC_024748) as outgroups. First, we aligned the 13 mitochondrial protein‐coding gene sequences in Clustal X 1.83 (Schattner et al., 2005) with the default settings, and then we concatenated individual genes excluding the stop codon. We selected the optimal nucleotide substitution model in jModeltest v0.1.1 (Posada, 2008) and used the Akaike Information Criterion (AIC: Posada & Buckley, 2004).

Maximum likelihood (ML) and Bayesian inference (BI) were used for phylogenetic analyses in MrBayes 3.2.2 (Ronquist et al., 2012), and BI of nucleotide acid datasets was performed using the GTR + I + G model (Lanave, Preparata, Saccone, & Serio, 1984). A ML tree was constructed using RAxML, and the robustness of the phylogenetic results was tested through bootstrap analysis with 1,000 replicates (Stamatakis, 2014).

## 3 RESULTS AND DISCUSSION

### 3.1 Genome content and organization

**Figure 2:**
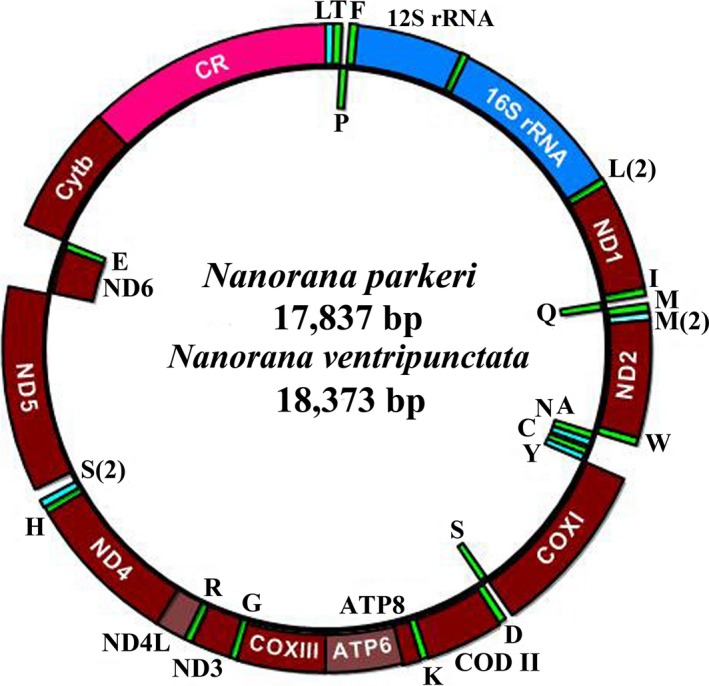
Complete mitochondrial genome organization and gene arrangement of *Nanorana ventripunctata* and *Nanorana pleskei*. Genes coded on the H strand are directed to the outer ring, while the genes coded on the L‐strand are indicated in the interior of the ring. Genes are abbreviated as follows: ATP6 and ATP8 (subunits 6 and 8 of ATPase), COXI‐COXIII (cytochrome c oxidase subunits 1–3), Cytb (cytochrome b), ND1‐ND6 and ND4L (NADH dehydrogenase subunits 1–6 and 4L), 12S rRNA and 16S rRNA (ribosomal RNA of 12S and 16S), CR (control region; noncoding region). One‐letter amino acid abbreviations were used to label the corresponding tRNA genes

The mitochondrial genomes of *N. parkeri* (17,837 bp), *N. ventripunctata* (18,373 bp) and *N. pleskei* (17,660 bp) included 13 PCGs (ND1‐6, ND4L, COX1‐3, Cyt *b*, ATP6 and ATP8), two ribosomal RNA genes (12S and 16S rRNA), 23 tRNA genes and one large noncoding region (putative control region; CR) (Table 1; Figure 2). In the three genomes, 12 protein‐coding genes (ND1‐5, ND4L, COX1‐3, ATP8, ATP6, and Cyt b) and two rRNAs (12S and 16S rRNA) were encoded on the heavy (H) strand along with 15 tRNAs (tRNA‐Leu1, tRNA‐Thr, tRNA‐Phe, tRNA‐Val, tRNA‐Leu2, tRNA‐Ile, tRNA‐Met1, tRNA‐Met2, tRNA‐Trp, tRNA‐Asp, tRNA‐Lys, tRNA‐Gly, tRNA‐Arg, tRNA‐His, and tRNA‐Ser2) and CR, while the remaining one protein‐coding gene (ND6) and eight tRNAs (tRNA‐Pro, tRNA‐Gln, tRNA‐Ala, tRNA‐Asn, tRNA‐Cys, tRNA‐Tyr, tRNA‐Ser1, and tRNA‐Glu) were encoded on the light (L) strand (Table 1; Figure 2). Overall we found that there were no differences in the gene arrangement of mitochondrial genomes among these *Nanorana* and *Quasipaa* species (Chen, Zhai, Zhu, & Chen, 2015; Simon et al., 1994; Zhou et al., 2009), but there were some differences between these two frog species and the typical neobatrachian type (e.g., *Rana nigromaculata*) in the position of tRNA‐Met, with formation of a tandem duplication of tRNA‐Met gene between tRNA‐Gln and ND2.

**Table 1:** The mitochondrial genome profile of three *Nanorana* species (*Nanorana parkeri*,* Nanorana ventripunctata*, and *Nanorana pleskei*)

Gene	Strand^a^	*Nanorana parkeri* (17,837 bp)						*Nanorana ventripunctata* (18,373 bp)						Nanorana pleskei (17,660 bp)					
		Position		Size	Codon		Intergenic bp^b^	Position		Size	Codon		Intergenic bp^b^	Position		Size	Codon		Intergenic bp^b^
		From	To		Start	Stop^c^		From	To		Start	Stop^c^		From	To		Start	Stop^c^	
tRNA‐Leu1	H	1	72	72			−3	1	72	72			0	1	81	81			−9
tRNA‐Thr	H	70	142	73			0	73	142	70			0	73	140	68			0
tRNA‐Pro	L	143	211	69			−1	143	211	69			−1	141	209	69			−1
tRNA‐Phe	H	211	280	70			0	211	280	70			0	209	278	70			0
12S RNA	H	281	1,216	936			0	281	1,217	937			0	279	1,212	934			0
tRNA‐Val	H	1,217	1,286	67			0	1,218	1,287	70			0	1,213	1,282	70			0
16S RNA	H	1,287	2,873	1,587			0	1,288	2,880	1,593			0	1,283	2,873	1,591			0
tRNA‐Leu2	H	2,874	2,946	73			0	2,881	2,953	73			0	2,874	2,946	73			0
ND1	H	2,947	3,904	958	GTG	T–	0	2,954	3,911	958	GTG	T–	0	2,947	3,904	958	GTG	T–	0
tRNA‐Ile	H	3,905	3,975	71			0	3,912	3,982	71			0	3,905	3,975	71			−1
tRNA‐Gln	L	3,976	4,046	71			0	3,983	4,053	71			0	3,975	4,045	71			0
tRNA‐Met1	H	4,047	4,115	69			10	4,054	4,122	69			9	4,046	4,114	69			9
tRNA‐Met2	H	4,126	4,194	69			0	4,132	4,200	69			0	4,124	4,192	69			0
ND2	H	4,195	5,227	1,033	ATT	T–	0	4,201	5,233	1,033	ATT	T–	0	4,193	5,225	1,033	ATT	T–	0
tRNA‐Trp	H	5,228	5,297	70			0	5,234	5,303	70			0	5,226	5,295	70			0
tRNA‐Ala	L	5,298	5,367	70			2	5,304	5,373	70			2	5,296	5,365	70			2
tRNA‐Asn	L	5,370	5,442	73			0	5,376	5,448	73			0	5,368	5,440	73			0
rep_origin																			
L‐strand	L	5,443	5,471	29			0	5,449	5,477	29			0	5,441	5,470	30			0
tRNA‐Cys	L	5,472	5,536	65			0	5,478	5,542	65			0	5,471	5,536	66			0
tRNA‐Tyr	L	5,537	5,603	67			4	5,543	5,609	67			4	5,537	5,603	67			4
COXI	H	5,608	7,158	1,551	ATA	AGG	−9	5,614	7,164	1,551	ATA	AGG	−9	5,608	7,158	1,551	ATA	AGG	−9
tRNA‐Ser1	L	7,150	7,220	71			0	7,156	7,226	71			0	7,150	7,220	71			0
tRNA‐Asp	H	7,221	7,289	69			1	7,227	7,296	70			2	7,221	7,289	69			2
COXII	H	7,291	7,975	685	ATG	T–	0	7,299	7,983	685	ATG	T–	0	7,292	7,976	685	ATG	T–	0
tRNA‐Lys	H	7,976	8,045	70			3	7,984	8,053	70			1	7,977	8,046	70			2
ATP8	H	8,049	8,210	162	ATG	TAA	−7	8,055	8,216	162	ATG	TAA	−7	8,049	8,210	162	ATG	TAA	−7
ATP6	H	8,204	8,885	682	ATG	T–	0	8,210	8,891	682	ATG	T–	0	8,204	8,885	682	ATG	T–	0
COXIII	H	8,886	9,669	784	ATG	T–	0	8,892	9,675	784	ATG	T–	0	8,886	9,669	784	ATG	T–	0
tRNA‐Gly	H	9,670	9,738	69			0	9,676	9,744	69			0	9,670	9,738	69			0
ND3	H	9,739	10,096	358	GTG	T–	0	9,745	10,102	385	GTG	T–	0	9,739	10,096	358	GTG	T–	0
tRNA‐Arg	H	10,097	10,165	69			1	10,103	10,171	69			1	10,097	10,165	69			1
ND4L	H	10,167	10,451	285	ATG	TAA	−7	10,173	10,457	285	ATG	TAA	−7	10,167	10,451	285	ATG	TAA	−7
ND4	H	10,445	11,807	1,363	ATG	T–	0	10,451	11,813	1,363	ATG	T–	0	10,445	11,807	1,363	ATG	T–	0
tRNA‐His	H	11,808	11,875	68			0	11,814	11,813	69			0	11,808	11,876	69			0
tRNA‐Ser2	H	11,876	11,943	68			106	11,883	11,950	68			37	11,877	11,944	68			44
ND5	H	12,050	13,873	1824	ATG	TAA	−15	11,988	13,811	1824	ATG	TAA	−15	11,989	13,812	1824	ATG	TAA	−15
ND6	L	13,859	14,356	498	ATG	AGA	0	13,797	14,294	498	ATG	AGA	0	13,798	14,295	498	ATG	AGA	0
tRNA‐Glu	L	14,357	14,425	69			7	14,295	14,363	69			7	14,296	14,364	69			7
CYTB	H	14,433	15,578	1,146	ATG	TAG	0	14,371	15,516	1,146	ATG	TAG	0	14,372	15,517	1,146	ATG	TAG	0
Control region	H	15,579	17,837	2,259			1	15,517	18,373	2,857			0	15,518	17,660	2,143			0

Note.^a^H and L indicate genes transcribed on the heavy and light strands, respectively. ^b^Numbers correspond to the nucleotides separating adjacent genes, negative numbers indicate overlapping nucleotides. ^c^T represents incomplete stop codons.

The gene components were very loosely juxtaposed with 134/42 (*N. parkeri*) and 63/39 (*N. ventripunctata*) of gap/overlapping nucleotides, compared to that of *N. pleskei* (71/49; Table 1) (Simon et al., 1994). Although the overall A + T contents of 57.87% in *N. parkeri* and 59.1% in *N. ventripunctata* were relatively higher than that of *N. pleskei* (57.5%), those values are within the range (52.8%–62.74%) of Dicroglossidae (Supporting information Table S1). The nucleotide skew was highly similar among these mitochondrial genomes including that of *N. pleskei*, with only some exceptions found on COX2, ATP6, ATP8 and the putative control region (Table 2).

**Table 2:** AT/CG skews in the mitochondrial protein‐coding genes (PCGs), 2 rRNA genes, CR and the entire mitochondrial genome from three *Nanorana* species (*Nanorana parkeri*,* Nanorana ventripunctata*, and *Nanorana pleskei*). The bold values indicated significant differences between the 3 species

Gene	AT‐skew			CG‐skew		
	*N. parkeri*	*N. ventripunctata*	*N. pleskei*	*N. parkeri*	*N. ventripunctata*	*N. pleskei*
ND1	−0.130	−0.141	−0.131	−0.356	−0.305	−0.305
ND2	−0.042	−0.072	−0.069	−0.510	−0.407	−0.404
ND3	−0.316	−0.212	−0.264	−0.309	−0.370	−0.287
ND4	−0.122	−0.119	−0.139	−0.362	−0.354	−0.336
ND4L	−0.124	−0.148	−0.157	−0.362	−0.328	−0.308
ND5	−0.091	−0.070	−0.097	−0.266	−0.289	−0.261
ND6	−0.261	−0.289	−0.300	0.453	0.490	0.453
COX1	−0.136	−0.114	−0.118	−0.160	−0.181	−0.160
COX2	**−0.005**	**0.028**	**−0.022**	−0.223	−0.236	−0.175
COX3	−0.127	−0.148	−0.187	−0.256	−0.240	−0.225
ATP6	**0.262**	**−0.137**	**−0.137**	−0.455	−0.410	−0.424
ATP8	**0.056**	**0.050**	**0.000**	−0.481	−0.508	−0.448
Cytb	−0.060	−0.088	−0.110	−0.339	−0.316	−0.297
12S rRNA	0.138	0.108	0.145	−0.111	−0.131	−0.119
16S rRNA	0.144	0.136	0.145	−0.101	−0.091	−0.096
CR	**−0.104**	**−0.216**	**−0.050**	−0.208	−0.219	−0.155
13PCG	−0.107	−0.110	−0.120	−0.277	−0.270	−0.248
Overall	−0.042	−0.066	−0.043	−0.261	−0.259	−0.155

### 3.2 Protein‐coding genes (PCGs) and codon usage patterns

The inferred start/stop codons for protein‐coding genes of *N. parkeri*,* N. ventripunctata*, and *N. pleskei* are listed in Table 1. In three mitochondrial genomes, the protein‐coding genes were initiated by ATG, with the exceptions of COX1, ND1, ND2, and ND3 (Table 1). The open reading frame of ND1 and ND3 started with GTG, while that of COX1 and ND2 started with ATA and ATT, respectively. The canonical stop codon (TAA or TAG) can be found in four protein‐coding genes (ATP8, Cytb, ND4L, and ND5; Table 1), while COX1 and ND6 use AGG and AGA as the termination codon, respectively. The remaining seven (ATP6, COX2‐3, and ND1‐4) had incomplete T‐stop codons (Table 1), completed (TAA) by polyadenylation after transcription (Boore, 2001).

The relative synonymous codon usage (RSCU) values of the three species of *Nanorana* mitogenomes are shown in Table 3, Supporting information Tables S2–S4. The results demonstrate that synonymous codon usage has a distinct bias toward A or T for 13 PCGs. The codons AUU (5.03%–5.62%), UUU (3.92%–4.37%), GCC (3.84%–4.03%), and CUU (3.63%–3.79%) were the four most frequently used codons in the mitogenomes of our three species of *Nanorana*, accounting for 16.42%–17.81%. In addition to GCC codon, these codons were mainly composed of A or U nucleotides, indicating the highly biased usage of A and T nucleotides in the three species of *Nanorana* PCGs. Meanwhile, the most frequently represented amino acids in the three species of *Nanorana* mitochondrial proteins were Leu (16.27%–16.38%), Ala (8.24%–8.32%), Ile (7.95%–8.11%), and Phe (6.62%–6.78%), accounting for 39.08%–39.59%. The least frequently represented amino acid was Cys (0.74%–0.77%). Codon usage of PCGs showed a major bias of A + T content, which played a major role in the A + T bias of the entire mitogenome. Similar patterns with a strong T‐ or A‐bias in the wobble position have been found among other *Nanorana* species also. The RSCU analysis showed that codons with A or T (U) at the third position are mostly overused compared with other synonymous codons. Therefore, the codon usage can reveal nucleotide bias too. These data imply a high A + T content in the three *Nanorana* species. The bias toward the use of Ts over As, to the 13 PCGs, is more obvious in these three *Nanorana* mitogenomes with −0.080 to −0.100 AT skewness. Moreover, negative AT‐skew and GC‐skew were found in the third position, whereas both the first and second positions showed positive AT‐skew and negative GC‐skew in *N. parkeri* and *N. ventripunctata*. In contrast, the first, second and third positions showed negative AT‐skew and GC‐skew in *N. pleskei* (Supporting information Table S5).

**Table 3:** Codon usage pattern of the 13 mitochondrial protein‐coding genes from three *Nanorana* species (*Nanorana parkeri*,* Nanorana ventripunctata*, and *Nanorana pleskei*. The asterisks “*” indicate terminate codon.)

Amino acids	Codon	No.			Amino acids	Codon	No.			Amino acids	Codon	No.		
		Npar	Nven	Nple			Npar	Nven	Nple			Npar	Nven	Nple
Phe	UUU	156	148	165		CCA	58	61	62		AAG	15	18	23
	UUC	103	102	91		CCG	25	18	19	Asp	GAU	30	31	33
Leu	UUA	118	123	117	Thr	ACU	80	73	86		GAC	44	40	37
	UUG	26	30	44		ACC	97	95	82	Glu	GAA	61	57	62
	CUU	143	142	137		ACA	109	100	112		GAG	29	32	29
	CUC	137	134	130		ACG	13	18	8	Cys	UGU	11	17	15
	CUA	140	128	132	Ala	GCU	72	69	78		UGC	18	12	13
	CUG	50	58	58		GCC	152	148	145	Trp	UGA	84	88	86
Ile	AUU	190	204	212		GCA	69	69	73		UGG	25	22	25
	AUC	112	102	88		GCG	19	25	18	Arg	CGU	14	11	12
Met	AUA	121	125	119	Tyr	UAU	63	61	60		CGC	18	23	20
	AUG	58	60	62		UAC	43	47	50		CGA	35	33	31
Val	GUU	71	71	84		UAA*	3	3	3		CGG	7	10	10
	GUC	43	52	47		UAG*	1	1	1	Ser	AGU	25	23	25
	GUA	59	66	73	His	CAU	34	28	34		AGC	30	33	29
	GUG	38	24	21		CAC	67	73	67		AGA*	1	1	1
Ser	UCU	62	67	71	Gln	CAA	75	78	77		AGG*	1	1	1
	UCC	79	72	68		CAG	18	13	14	Gly	GGU	36	42	47
	UCA	70	78	78	Asn	AAU	63	69	63		GGC	74	69	61
	UCG	16	7	9		AAC	62	49	54		GGA	50	52	48
Pro	CCU	33	34	40	Lys	AAA	69	73	64		GGG	58	63	67
	CCC	91	98	83										

### 3.3 Transfer and ribosomal RNA genes

**Figure 3:**
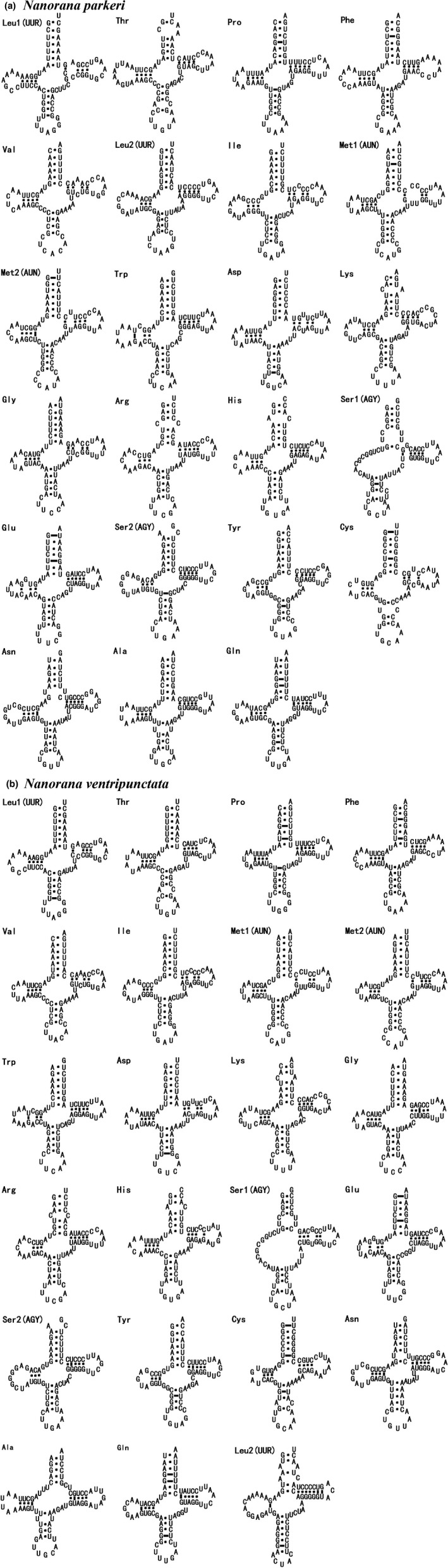
Putative tRNA secondary structures predicted from the 22 tRNA gene sequences found in the (a) *Nanorana parkeri* and (b) *Nanorana ventripunctata* mitochondrial genome

A total of 23 tRNA genes (including an extra copy of tRNA‐Met gene) with 65 to 73 bp in length were identified in mitochondrial genomes of both *N. parkeri* and *N. ventripunctata* (Figure 3), including an extra copy of the tRNA‐Met genes. All tRNA genes can fold into the canonical cloverleaf secondary structure with the same anticodon usage as reported in other vertebrates. The sequences, anticodon nucleotides, and secondary structures of tRNA genes in *N. parkeri* and *N. ventripunctata* were very similar to those in *N. pleskei* (Chen et al., 2011) (Figure 3). In addition, a tandem repeat of tRNA‐Met gene was easy to find in the three *Nanorana* species, located between the tRNA‐Gln and ND2 genes. Extra tRNA‐Met was also found in *Quasipaa boulengeri*,* Fejervarya cancrivora*,* Hoplobatrachus rugulosus*,* Euphlyctis hexadactylus*,* Limnonectes bannaensis*, and *Occidozyga martensii* (Alam et al., 2010; Chen et al., 2011; Li et al., 2014a, 2014b; Ren et al., 2009; Shan, Xia, Zheng, Zou, & Zeng, 2014; Zhang et al., 2009). But this phenomenon is different to that seen in *Amolops tormotus* and other typical vertebrates (Su, Wu, Yan, Cao, & Hu, 2007). Two tRNA‐Met genes in each lineage may come from different origins (Kurabayashi et al., 2006), and the tandem duplication of the tRNA‐Met gene can be seen as a synapomorphic feature of Dicroglossidae. A tandem duplication of the mitochondrial tRNA‐Pro and tRNA‐Thr genes in *Bipes biporus* has been reported from previous research (Macey, Schulte, Larson, & Papenfuss, 1998). Based on tandem duplication/deletion models, pseudogene formation in tandemly duplicated sequences might result from mtDNA rearrangement. Our results found two tandem tRNA‐Met genes in *N. parkeri* and *N. ventripunctata* which supports this view.

12S and 16S rRNAs were encoded on the H strand and were separated by tRNA‐Val in both of the mitochondrial genomes that we analyzed. The length of 12S and 16S rRNA genes was estimated to be 936 and 1,587 bp for *N. parkeri*, and 937 and 1,593 bp for *N. ventripunctata*, respectively.

### 3.4 Noncoding regions

Putative control region, of 2,259 bp (*N. parkeri*) and 2,857 bp (*N. ventripunctata*) were found in Cytb and tRNA‐Leu, which is longer than that of *N. pleskei* (2,143 bp) (Table 1). The size of control region variation demonstrated different lengths of the total mitogenomes for the three species. The A + T contents (65.96% in *N. parkeri* and 69.86% in *N. ventripunctata*) in control region were higher than in other regions (Table 1). Additionally, the A + T contents rated different lengths of the total mitogenomes for the three species. The A + T content in this region is higher than that in the coding regions (Boore, 1999; Simon et al., 1994). The L‐strand origin of replication (O_L_) of the *N. parkeri* and *N. ventripunctata* mitogenome is located at the gene boundary of tRNA‐Asn and tRNA‐Cys in the WANCY tRNA gene cluster and has the same sequence as *N. pleskei* (Chen et al., 2011). The sequence, structure, and position of O_L_ are well conserved in the anuran mitogenomes, and are also similar to those in other vertebrates (San Mauro, Gower, Zardoya, & Wilkinson, 2006), implying it is a common and important characteristic of this short intergenic spacer region.

**Figure 4:**
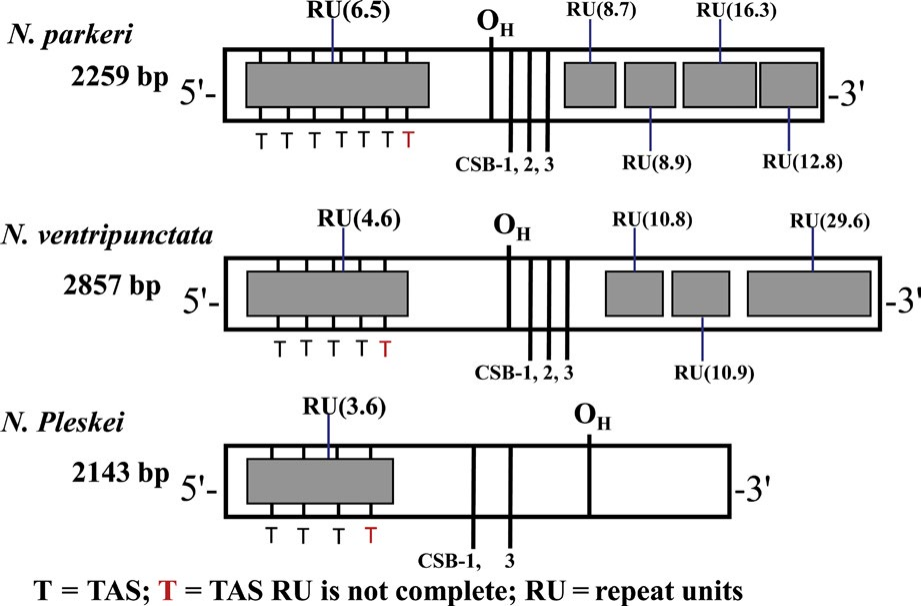
Main features of three *Nanorana* species control region. The location of features is shown in Table 4

**Figure 5:**
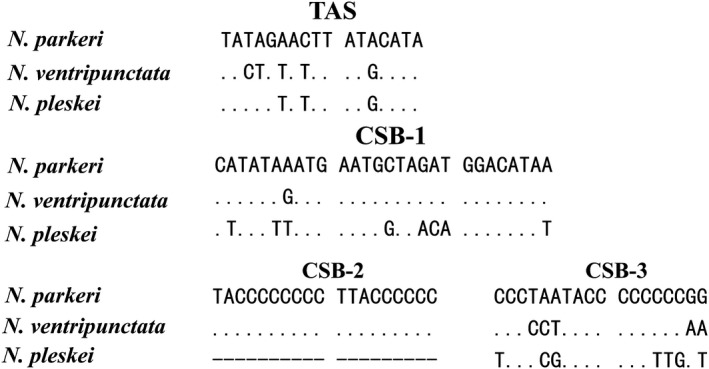
Structures and alignments of identified putative termination‐associated sequences (TAS) and, conserved sequence blocks (CSB 1‐3). Alignment gaps and nucleotides identical to the first line are indicated by dashes (–) and a dot (∙), respectively. Variable nucleotides are marked with corresponding nucleotides

On both 5′ and 3′‐sides, the control region has distinct repeat regions (Figure 4) with the 5′‐side repeat region consisting of 6.5 and 4.6 tandem repeat units of 124 bp in *N. ventripunctata* and *N. parkeri*, respectively. Dissimilar to the 5′‐side repeat region, the 3′‐side repeat region includes 10.8 tandem repeat units of 11 bp (5′‐GCTCGTATTCT‐3′), 10.9 tandem repeat units of 11 bp (5′‐CTTCGCTTATC‐3′), 29.6 tandem repeat units of 10 bp (5′‐GTTTTTGTTA‐3′), 8.7 tandem repeat units of 11 bp (5′‐GCTCGTATATT‐3′), 8.9 tandem repeat units of 11 bp (5′‐ATACTTCGCTT‐3′), 16.3 tandem repeat units of 8 bp (5′‐TAATTGTA‐3′), 12.8 tandem repeat units of 8 bp (5′‐GCTGATCG‐3′), respectively (Table 4). No tandem repeats in the 3′‐side region were found in *N. pleskei* which is unusual for a mitogenome control region. Unlike the corresponding region in other anurans, the control region of *N. ventripunctata* and *N. parkeri* included TAS, CSB‐1, CSB‐2, CSB‐3, and O_H_ (Figure 5). The 5′‐side tandem repeat units included 6.5 and 4.6 putative termination‐associated sequences (TASs, 5′‐TATAAGACATCTAT GTA‐3′) of *N. ventripunctata* and *N. parkeri*, respectively (Table 4). Tandem repeat units including TASs were also detected in the control regions of *Bufo japonicas*,* Paa spinosa* and *Hyla japonica* (Igawa, Kurabayashi, Usuki, Fujii, & Sumida, 2008; Zhou et al., 2009). Three conserved sequence blocks (CSBs) may be related to in the initiation of the mtDNA synthesis and they (CSB‐1, CSB‐2, CSB‐3) can be identified between the tandem repeat units at the 5′ and 3′‐sides (Table 4; Figure 4). CSB‐1, CSB‐2 and CSB‐3 of *N. ventripunctata* and *N. parkeri* showed high similarity to the consensus in other amphibians, while the variation in *N. pleskei* is slightly larger (Figure 5); moreover, CSB‐1 is not reduced to a truncated penta motif (5′‐GACAT‐3′) as it is in the caecilians (San Mauro et al., 2004; Zardoya & Meyer, 2000). However, a truncated CSB‐1 had been recorded in *Xenopus laevis* (Anura) (Roe, Ma, Wilson, & Wong, 1985). The CSB‐2 motif was not found in *N. pleskei* (Figure 5). In addition, the multiple motifs of mtDNA control regions (CR) may be associated with the transcription and replication of the mitochondrial genome (Taanman, 1999). The function of these conserved sequence blocks is unclear. Further study on the mechanistic basis of mtDNA replication is warranted for *Nanorana* species.

**Table 4:** Location of features in the D‐loop of three *Nanorana* species (*Nanorana parkeri*,* Nanorana ventripunctata*, and *Nanorana pleskei*)

Species	Sequence in D‐loop	Start position	Stop position	Length (bp)
*Nanorana ventripunctata*	6.5 tandem repeat units	21	830	5 × 124 + 66
	TAS	119	135	17
	TAS	243	259	17
	TAS	367	383	17
	TAS	491	507	17
	TAS	615	631	17
	TAS	739	755	17
	OH	1,552	1,623	72
	CSB‐1	1,713	1,740	28
	CSB‐2	1809	1827	19
	CSB‐3	1814	1831	18
	10.8 tandem repeat units	1917	2035	10 × 11 + 9
	10.9 tandem repeat units	2042	2,161	10 × 11 + 10
	29.6 tandem repeat units	2,196	2,492	29 × 10 + 6
*Nanorana parkeri*	4.6 tandem repeat units	42	610	4 × 124 + 72
	TAS	151	167	17
	TAS	275	291	17
	TAS	399	415	17
	TAS	523	539	17
	OH	1,389	1,460	72
	CSB‐1	1,495	1,522	28
	CSB‐2	1,595	1,613	19
	CSB‐3	1,640	1,657	18
	8.7 tandem repeat units	1,702	1,796	8 × 11 + 8
	8.9 tandem repeat units	1801	1898	8 × 11 + 10
	16.3 tandem repeat units	1898	2027	16 × 8 + 10
	12.8 tandem repeat units	2028	2,129	12 × 8 + 8
*Nanorana pleskei*	3.6 tandem repeat units	22	467	3 × 124 + 74
	TAS	130	146	17
	TAS	254	270	17
	TAS	378	394	17
	OH	1,554	1,627	74
	CSB‐1	514	541	28
	CSB‐3	1,203	1,220	18

### 3.5 Phylogenetic relationships

The concatenated PCG data of the mitogenome sequences in our study contained 11,292 nucleotide positions, including 4,314 conserved sites, 6,978 variable sites and 6,505 potentially parsimony‐informative sites. Phylogenetic trees were reconstructed using BI and ML analyses, based on the nucleotide dataset. The use of PCG sequences of the mitogenomes has become an informative strategy for inferring phylogenetic relationships (Boore, Macey, & Medina, 2005). Using the 13 PCG sequences to concatenate may achieve a more complete analysis. BI and ML methods consistently support similar tree topologies by strong node‐supporting values.

**Figure 6:**
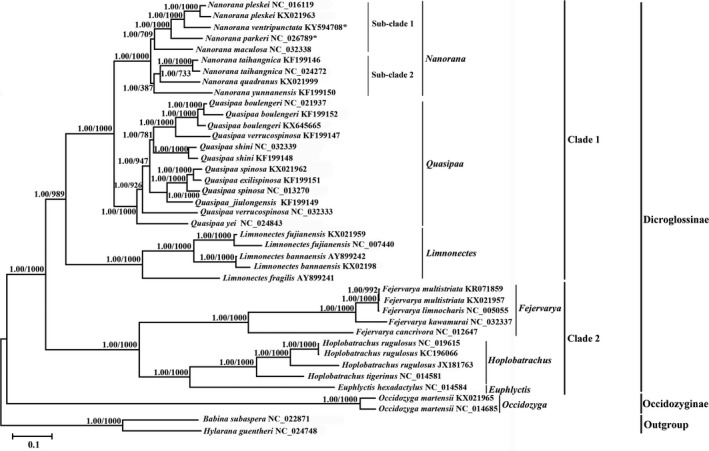
Results of phylogenetic analyses using BI and ML analysis indicated evolutionary relationships among 38 individuals based on 13 PCGs sequences. *Babina subaspera* (NC_022871) and *Hylarana guentheri* (NC_024748) were used as outgroups. Tree topologies produced by BI and ML analyses were equivalent. Bayesian posterior probability (PP) and bootstrap support (BP) values for ML analyses are shown in order on the nodes. The asterisks indicate new sequences generated in this study

So far, combined with the 38 mitochondrial genome sequences in GenBank database, our phylogenetic analyses revealed that the subfamily Dicroglossinae's monophyly was well supported (Li et al., 2014a, 2014b; Roelants et al., 2007; Yuan et al., 2016). The subfamily Dicroglossinae is the sister clade to the Occidozyginae (Figure 6). The Dicroglossinae species was divided into two clades with one clade (Clade 1) containing *Nanorana*,* Quasipaa*, and *Limnonectes*, and the other (Clade 2) including *Fejervarya*,* Euphlyctis*, and *Hoplobatrachus* (Figure 6), as supported by previous studies (Lv, Bi, & Fu, 2014; Yuan et al., 2016; Zhang, Xia, & Zeng, 2016). *Quasipaa* and *Nanorana* belong to the sister genus. Clade *Quasipaa* comprised *Q. yei* as the sister taxon to the subclade (BP = 100%, PP = 1.00) containing (((*Q. jiulongensis* + (*Q. spinosa* + *Q. exilispinosa*)) + (*Q. shini* + (*Q. boulengeri* + *Q. verrucospinosa*)))) (Figure 6). Within the genus *Quasipaa*, the phylogenetic inferences based on mtDNA sequences showed that all individuals of *Q. boulengeri* formed a monophyly with high support, sister to *Q. verruspinosa* (KF199147). This result is similar to the results from Che et al. (2009), but different to those of Qing et al. (2012). Furthermore, we find that *Q. verruspinosa* is paraphyletic in the genus *Quasipaa*, as one sample of *Q. verruspinosa* (KF19917) was grouped with *Q. boulengeri*, while another sample of *Q. verruspinosa* (NC_032333) was not (Figure 6). All the *Nanorana* species were clustered together. In the *Nanorana* clade, *N. yunnanensis* is the sister group of a clade composed of *N. quadranus* and *N. taihangnica* (Subclade 2). And the Subclade 2 composed of these 3 species is the sister group of a clade (Subclade 1) that includes *N. pleskei*,* N. ventripunctata*,* N. parkeri* and *N. maculosa*. So the *Nanorana* species clustered in a single monophyly. Our molecular phylogeny indicates *N. ventripunctata* and *N. pleskei* are more closely related compared with *N. parkeri*, and strongly supports that *N. parkeri* is basal to *N. pleskei* and *N. ventripunctata* based on 13 PCG genes of the mitogenome (BP = 100%, PP = 1.00) (Figure 6), in agreement with the relationships inferred by the research report of Che et al. (2009, 2010). However the phylogeny of the three species (*N. parkeri*,* N. pleskei* and *N. ventripunctata*) based on 13 PCGs was not concordant with those reported earlier based on 12 genes (three mitochondrial and nine nuclear genes) (Pyron & Wiens, 2011). This difference may be caused by the use of different molecular markers, and their evolutionary relationships need further investigating and searching for more evidences from molecular markers and morphological characters. *Nanorana* and *Quasipaa* were resolved as the sister group of the genus *Limnonectes* (BP = 99%, PP = 1.00). The phylogenetic relationships supported the authenticity of the two obtained mitogenomes among *Nanorana*. And the phylogenetic reconstruction using the whole mitogenome, rather than single genes, provided more credible results. The mitogenomic approach, as previously demonstrated (Cai, Che, Pang, Zhao, & Zhang, 2007; Liyan, Xia, Zheng, & Zeng, 2012; Yan et al., 2013), is an excellent tool with which to infer phylogenetic relationships within Neobatrachia. In the present study, all clades were well resolved, with only a few exceptions less than 90%, while Bayesian posterior probabilities were 1.00. Despite their fast evolutionary rates, mitochondrial genomes contain species‐specific evolutionary affinities, which can be efficiently recovered by improving taxon sampling (Rubinstein et al., 2013).

## 4 CONCLUSIONS

In summary, the complete mitochondrial genomes of two *Nanorana* species were determined in this study. Our mitogenome analyses, including gene content, gene order, strand asymmetry, base composition, rRNA and tRNA secondary structure and phylogenetic analysis, indicate several significant features: a tandem repeat of the tRNA‐Met gene was detected in three *Nanorana* species, located between the tRNA‐Gln and ND2 genes. The control region contains distinct repeat regions at both 5′ and 3′‐sides, and the CSB‐2 motif was not found in the *N. pleskei*. Based on nucleotide sequences of 13 PCGs, and using BI and maximum‐likelihood analyses, the phylogenetic data illustrate the taxonomic status of *Nanorana* and provides robust support that *N. ventripunctata* and *N. pleskei* are more closely related than *N. parkeri*. Our study provides useful additional data for further phylogenetic analysis of the *Nanorana* genus. Expanding our knowledge of the phylogenetic relationships within the *Nanorana* genus will ultimately aid in future research to protect and maintain biodiversity within many other anuran species. However, the proposed evolutionary relationships among these three species based on the findings that emerged in the study should be accepted with caution due to limited taxon sampling. Many aspects of the phylogeny of the genus *Nanorana* remain to be resolved and further analysis based on more molecular information (including nuclear gene data) and extensive taxon sampling is necessary to elucidate the phylogenetic relationships among genus *Nanorana* or Dicroglossidae.

## CONFLICT OF INTEREST

The authors have declared that no competing interest exists.

## AUTHOR CONTRIBUTION

Jiang L., Ruan Q., and Chen W. designed the manuscript, You Z. and Yu P. analyzed the data, and Jiang L. and Chen W. wrote the manuscript.
