# Supplementary material for: The first complete mitochondrial genome sequence of Nanorana parkeri and Nanorana ventripunctata (Amphibia: Anura: Dicroglossidae), with related phylogenetic analyses
Source: Ecol Evol. 2018 Jun 11;8(14):6972–87. doi: 10.1002/ece3.4214 (PMC6065340; doi:10.1002/ece3.4214)
Supplement: Supplementary file 1 [file ECE3-8-6972-s001.docx]

**Table S1** Comparison of whole genome sizes and A + T contents, and GenBank accession numbers of the mitochondrial genomesfor 40 fork-tongued frogs.

| Species | Total | | 13 PCGs | | 16S rRNA gene | | 12S rRNA gene | | CR | | Accession Numbers |
| --- | --- | --- | --- | --- | --- | --- | --- | --- | --- | --- | --- |
|  | Length (bp) | A+T (%) | Length (bp) | A+T (%) | Length (bp) | A+T (%) | Length (bp) | A+T (%) | Length (bp) | A+T (%) |  |
| *Nanorana ventripunctata* | 18373 | 59.10 | 11329 | 56.43 | 1593 | 60.80 | 937 | 54.80 | 2857 | 69.86 | KY594708 |
| *Nanorana parkeri* | 17837 | 57.87 | 11329 | 56.30 | 1587 | 59.61 | 936 | 54.06 | 2259 | 65.96 | NC_026789 |
| *Nanorana pleskei* | 17660 | 57.50 | 11329 | 57.20 | 1591 | 60.80 | 934 | 54.80 | 2143 | 56.20 | NC_016119 |
| *Nanorana pleskei* | 19094 | 61.09 | 11322 | 57.25 | 1590 | 61.13 | 933 | 54.98 | / | / | KX021963 |
| *Nanorana taihangnica* | 17412 | 57.21 | 11308 | 56.22 | 1586 | 58.95 | 935 | 55.29 | 1972 | 61.82 | NC_024272 |
| *Nanorana taihangnica* | 21322 | 57.87 | 11311 | 56.19 | 1585 | 58.99 | 934 | 55.57 | 2014 | 61.62 | KF199146 |
| *Nanorana delacouri* | 17452 | 60.46 | 11310 | 59.24 | 1585 | 60.95 | 932 | 54.40 | / | / | NC_032305 |
| *Nanorana maculosa* | 18445 | 59.78 | 11310 | 58.12 | 1589 | 60.73 | 934 | 54.60 | / | / | NC_032338 |
| *Nanorana yunnanensis* | 14521 | 58.08 | 10483 | 57.76 | 1582 | 60.75 | 917 | 55.51 | / | / | KF199150 |
| *Nanorana yunnanensis* | 8305 | 58.21 | 11311 | 56.19 | 1583 | 60.90 | 502 | 55.38 | / | / | KX021997 |
| *Nanorana quadranus* | 15448 | 57.68 | 10818 | 56.36 | 1009 | 61.25 | / | / | / | / | KX021999 |
| *Quasipaa yei* | 17072 | 58.00 | 11308 | 56.86 | 1582 | 60.43 | 932 | 54.51 | 1580 | 66.96 | NC_024843 |
| *Quasipaa boulengeri* | 17741 | 59.48 | 11308 | 58.50 | 1578 | 60.33 | 931 | 53.60 | 2047 | 67.95 | NC_021937 |
| *Quasipaa boulengeri* | 15106 | 58.41 | 11308 | 56.86 | 1580 | 60.44 | 921 | 53.64 | / | / | KF199152 |
| *Quasipaa boulengeri* | 16672 | 58.64 | 11359 | 58.16 | 1582 | 60.24 | 933 | 54.02 | 648 | 74.54 | KX645665 |
| *Quasipaa spinosa* | 18012 | 59.66 | 11308 | 58.64 | 1584 | 59.91 | 934 | 54.18 | 2524 | 66.80 | NC_013270 |
| *Quasipaa spinosa* | 17704 | 58.86 | 11313 | 57.92 | 1589 | 59.66 | 934 | 54.39 | / | / | KX021962 |
| *Quasipaa jiulongensis* | 15072 | 58.52 | 11020 | 58.68 | 1583 | 60.01 | 922 | 55.10 | / | / | KF199149 |
| *Quasipaa exilispinosa* | 14903 | 58.02 | 11018 | 57.94 | 1583 | 59.76 | 934 | 54.82 | / | / | KF199151 |
| *Quasipaa shini* | 17549 | 59.73 | 11288 | 58.68 | 1577 | 59.99 | 931 | 55.32 | / | / | NC_032339 |
| *Quasipaa shini* | 14943 | 58.44 | 11785 | 58.24 | 1576 | 59.77 | 932 | 55.26 | / | / | KF199148 |
| *Quasipaa verrucospinosa* | 17476 | 62.74 | 11310 | 61.80 | 1586 | 62.67 | 931 | 57.04 | 2766 | 65.91 | NC_032333 |
| *Quasipaa verrucospinosa* | 15063 | 58.60 | 11029 | 58.84 | 1584 | 60.80 | 895 | 53.30 | / | / | KF199147 |
| *Limnonectes bannaensis* | 16867 | 58.59 | 11407 | 58.49 | 1586 | 59.52 | 928 | 54.53 | 1186 | 60.12 | AY899242 |
| *Limnonectes bannaensis* | 16862 | 58.64 | 11379 | 58.61 | 1580 | 59.56 | 929 | 54.47 | / | / | KX021984 |
| *Limnonectes fragilis* | 16640 | 57.94 | 11298 | 58.05 | 1579 | 58.58 | 924 | 53.35 | 1322 | 61.57 | AY899241 |
| *Limnonectes fujianensis* | 17654 | 57.60 | 11371 | 56.95 | 1581 | 59.84 | 925 | 53.95 | 1577 | 62.33 | NC_007440 |
| *Limnonectes fujianensis* | 18057 | 57.51 | 11501 | 57.18 | 1577 | 59.67 | 928 | 53.56 | / | / | KX021959 |
| *Fejervarya cancrivora* | 17843 | 56.73 | 11253 | 56.46 | 1429 | 57.31 | 933 | 54.13 | 2441 | 59.12 | NC_012647 |
| *Fejervarya limnocharis* | 17717 | 58.02 | 11241 | 57.13 | 1587 | 59.61 | 933 | 55.95 | 2180 | 63.39 | NC_005055 |
| *Fejervarya kawamurai* | 17500 | 56.85 | 11886 | 56.32 | 1593 | 58.95 | 933 | 55.20 | / | / | NC_032337 |
| *Fejervarya multistriata* | 17750 | 57.88 | 11241 | 57.14 | 1590 | 59.62 | 933 | 55.73 | 2212 | 62.25 | KR071859 |
| *Fejervarya multistriata* | 17243 | 57.83 | 11257 | 57.13 | 1590 | 59.62 | 933 | 55.73 | / | / | KX021957 |
| *Hoplobatrachus rugulosus* | 20926 | 52.80 | 11318 | 51.09 | / | / | 940 | 51.60 | 1815 | 56.97 | JX181763 |
| *Hoplobatrachus rugulosus* | 16903 | 53.03 | 11327 | 52.15 | 1583 | 57.23 | 937 | 52.08 | 1494 | 54.02 | KC196066 |
| *Hoplobatrachus rugulosus* | 20309 | 53.03 | 11303 | 52.13 | 1586 | 57.19 | 936 | 52.14 | 1496 | 54.21 | NC_019615 |
| *Hoplobatrachus tigerinus* | 20462 | 55.49 | 11299 | 55.20 | 1586 | 59.21 | 934 | 52.36 | 3415 | 54.90 | NC_014581 |
| *Euphlyctis hexadactylus* | 20280 | 56.99 | 11309 | 57.01 | 1584 | 58.71 | 938 | 53.41 | 2648 | 57.06 | NC_014584 |
| *Occidozyga martensii* | 18321 | 62.26 | 11280 | 61.84 | 1581 | 62.56 | 939 | 57.83 | 2766 | 65.91 | NC_014685 |
| *Occidozyga martensii* | 18929 | 61.57 | 11239 | 61.05 | 1581 | 61.48 | 940 | 57.45 | / | / | KX021965 |

**TABLE S2** Codon usage in *Nanorana parkeri* mitochondrial protein-coding genes

| Codon | Count | RSCU | % | Codon | Count | RSCU | % | Codon | Count | RSCU | % | Codon | Count | RSCU | % |
| --- | --- | --- | --- | --- | --- | --- | --- | --- | --- | --- | --- | --- | --- | --- | --- |
| UUU(F) | **156** | 1.2 | **4.13** | UCU(S) | 62 | 1.32 | 1.64 | UAU(Y) | 63 | 1.19 | 1.67 | UGU(C) | 11 | 0.76 | 0.29 |
| UUC(F) | 103 | 0.8 | 2.73 | UCC(S) | 79 | 1.68 | 2.09 | UAC(Y) | 43 | 0.81 | 1.14 | UGC(C) | 18 | 1.24 | 0.48 |
| UUA(L) | 118 | 1.15 | 3.13 | UCA(S) | 70 | 1.49 | 1.85 | UAA(*) | 3 | 2 | 0.08 | UGA(W) | 84 | 1.54 | 2.23 |
| UUG(L) | 26 | 0.25 | 0.69 | UCG(S) | 16 | 0.34 | 0.42 | UAG(*) | 1 | 0.67 | 0.03 | UGG(W) | 25 | 0.46 | 0.66 |
| CUU(L) | **143** | 1.4 | **3.79** | CCU(P) | 33 | 0.64 | 0.87 | CAU(H) | 34 | 0.67 | 0.90 | CGU(R) | 14 | 0.76 | 0.37 |
| CUC(L) | 137 | 1.34 | 3.63 | CCC(P) | 91 | 1.76 | 2.41 | CAC(H) | 67 | 1.33 | 1.78 | CGC(R) | 18 | 0.97 | 0.48 |
| CUA(L) | 140 | 1.37 | 3.71 | CCA(P) | 58 | 1.12 | 1.54 | CAA(Q) | 75 | 1.61 | 1.99 | CGA(R) | 35 | 1.89 | 0.93 |
| CUG(L) | 50 | 0.49 | 1.32 | CCG(P) | 25 | 0.48 | 0.66 | CAG(Q) | 18 | 0.39 | 0.48 | CGG(R) | 7 | 0.38 | 0.19 |
| AUU(I) | **190** | 1.26 | **5.03** | ACU(T) | 80 | 1.07 | 2.12 | AAU(N) | 63 | 1.01 | 1.67 | AGU(S) | 25 | 0.53 | 0.66 |
| AUC(I) | 112 | 0.74 | 2.97 | ACC(T) | 97 | 1.3 | 2.57 | AAC(N) | 62 | 0.99 | 1.64 | AGC(S) | 30 | 0.64 | 0.79 |
| AUA(M) | 121 | 1.35 | 3.21 | ACA(T) | 109 | 1.46 | 2.89 | AAA(K) | 69 | 1.64 | 1.83 | AGA(*) | 1 | 0.67 | 0.03 |
| AUG(M) | 58 | 0.65 | 1.54 | ACG(T) | 13 | 0.17 | 0.34 | AAG(K) | 15 | 0.36 | 0.40 | AGG(*) | 1 | 0.67 | 0.03 |
| GUU(V) | 71 | 1.35 | 1.88 | GCU(A) | 72 | 0.92 | 1.91 | GAU(D) | 30 | 0.81 | 0.79 | GGU(G) | 36 | 0.66 | 0.95 |
| GUC(V) | 43 | 0.82 | 1.14 | GCC(A) | **152** | 1.95 | **4.03** | GAC(D) | 44 | 1.19 | 1.17 | GGC(G) | 74 | 1.36 | 1.96 |
| GUA(V) | 59 | 1.12 | 1.56 | GCA(A) | 69 | 0.88 | 1.83 | GAA(E) | 61 | 1.36 | 1.62 | GGA(G) | 50 | 0.92 | 1.32 |
| GUG(V) | 38 | 0.72 | 1.01 | GCG(A) | 19 | 0.24 | 0.50 | GAG(E) | 29 | 0.64 | 0.77 | GGG(G) | 58 | 1.06 | 1.54 |

**TABLE S3** Codon usage in *Nanorana ventripunctata* mitochondrial protein-coding genes

| Codon | Count | RSCU | % | Codon | Count | RSCU | % | Codon | Count | RSCU | % | Codon | Count | RSCU | % |
| --- | --- | --- | --- | --- | --- | --- | --- | --- | --- | --- | --- | --- | --- | --- | --- |
| UUU(F) | **148** | 1.18 | **3.92** | UCU(S) | 67 | 1.44 | 1.78 | UAU(Y) | 61 | 1.13 | 1.62 | UGU(C) | 17 | 1.17 | 0.45 |
| UUC(F) | 102 | 0.82 | 2.70 | UCC(S) | 72 | 1.54 | 1.91 | UAC(Y) | 47 | 0.87 | 1.25 | UGC(C) | 12 | 0.83 | 0.32 |
| UUA(L) | 123 | 1.2 | 3.26 | UCA(S) | 78 | 1.67 | 2.07 | UAA(*) | 3 | 2 | 0.08 | UGA(W) | 88 | 1.6 | 2.33 |
| UUG(L) | 30 | 0.29 | 0.79 | UCG(S) | 7 | 0.15 | 0.19 | UAG(*) | 1 | 0.67 | 0.03 | UGG(W) | 22 | 0.4 | 0.58 |
| CUU(L) | **142** | 1.39 | **3.76** | CCU(P) | 34 | 0.64 | 0.90 | CAU(H) | 28 | 0.55 | 0.74 | CGU(R) | 11 | 0.57 | 0.29 |
| CUC(L) | 134 | 1.31 | 3.55 | CCC(P) | 98 | 1.86 | 2.60 | CAC(H) | 73 | 1.45 | 1.93 | CGC(R) | 23 | 1.19 | 0.61 |
| CUA(L) | 128 | 1.25 | 3.39 | CCA(P) | 61 | 1.16 | 1.62 | CAA(Q) | 78 | 1.71 | 2.07 | CGA(R) | 33 | 1.71 | 0.87 |
| CUG(L) | 58 | 0.57 | 1.54 | CCG(P) | 18 | 0.34 | 0.48 | CAG(Q) | 13 | 0.29 | 0.34 | CGG(R) | 10 | 0.52 | 0.26 |
| AUU(I) | **204** | 1.33 | **5.41** | ACU(T) | 73 | 1.02 | 1.93 | AAU(N) | 69 | 1.17 | 1.83 | AGU(S) | 23 | 0.49 | 0.61 |
| AUC(I) | 102 | 0.67 | 2.70 | ACC(T) | 95 | 1.33 | 2.52 | AAC(N) | 49 | 0.83 | 1.30 | AGC(S) | 33 | 0.71 | 0.87 |
| AUA(M) | 125 | 1.35 | 3.31 | ACA(T) | 100 | 1.4 | 2.65 | AAA(K) | 73 | 1.6 | 1.93 | AGA(*) | 1 | 0.67 | 0.03 |
| AUG(M) | 60 | 0.65 | 1.59 | ACG(T) | 18 | 0.25 | 0.48 | AAG(K) | 18 | 0.4 | 0.48 | AGG(*) | 1 | 0.67 | 0.03 |
| GUU(V) | 71 | 1.33 | 1.88 | GCU(A) | 69 | 0.89 | 1.83 | GAU(D) | 31 | 0.87 | 0.82 | GGU(G) | 42 | 0.74 | 1.11 |
| GUC(V) | 52 | 0.98 | 1.38 | GCC(A) | **148** | 1.9 | **3.92** | GAC(D) | 40 | 1.13 | 1.06 | GGC(G) | 69 | 1.22 | 1.83 |
| GUA(V) | 66 | 1.24 | 1.75 | GCA(A) | 69 | 0.89 | 1.83 | GAA(E) | 57 | 1.28 | 1.51 | GGA(G) | 52 | 0.92 | 1.38 |
| GUG(V) | 24 | 0.45 | 0.64 | GCG(A) | 25 | 0.32 | 0.66 | GAG(E) | 32 | 0.72 | 0.85 | GGG(G) | 63 | 1.12 | 1.67 |

**TABLE S4** Codon usage in *Nanorana pleskei* mitochondrial protein-coding genes

| Codon | Count | RSCU | % | Codon | Count | RSCU | % | Codon | Count | RSCU | % | Codon | Count | RSCU | % |
| --- | --- | --- | --- | --- | --- | --- | --- | --- | --- | --- | --- | --- | --- | --- | --- |
| UUU(F) | **165** | 1.29 | **4.37** | UCU(S) | 71 | 1.52 | 1.88 | UAU(Y) | 60 | 1.09 | 1.59 | UGU(C) | 15 | 1.07 | 0.40 |
| UUC(F) | 91 | 0.71 | 2.41 | UCC(S) | 68 | 1.46 | 1.80 | UAC(Y) | 50 | 0.91 | 1.32 | UGC(C) | 13 | 0.93 | 0.34 |
| UUA(L) | 117 | 1.14 | 3.10 | UCA(S) | 78 | 1.67 | 2.07 | UAA(*) | 3 | 2 | 0.08 | UGA(W) | 86 | 1.55 | 2.28 |
| UUG(L) | 44 | 0.43 | 1.17 | UCG(S) | 9 | 0.19 | 0.24 | UAG(*) | 1 | 0.67 | 0.03 | UGG(W) | 25 | 0.45 | 0.66 |
| CUU(L) | **137** | 1.33 | **3.63** | CCU(P) | 40 | 0.78 | 1.06 | CAU(H) | 34 | 0.67 | 0.90 | CGU(R) | 12 | 0.66 | 0.32 |
| CUC(L) | 130 | 1.26 | 3.44 | CCC(P) | 83 | 1.63 | 2.20 | CAC(H) | 67 | 1.33 | 1.78 | CGC(R) | 20 | 1.1 | 0.53 |
| CUA(L) | 132 | 1.28 | 3.50 | CCA(P) | 62 | 1.22 | 1.64 | CAA(Q) | 77 | 1.69 | 2.04 | CGA(R) | 31 | 1.7 | 0.82 |
| CUG(L) | 58 | 0.56 | 1.54 | CCG(P) | 19 | 0.37 | 0.50 | CAG(Q) | 14 | 0.31 | 0.37 | CGG(R) | 10 | 0.55 | 0.26 |
| AUU(I) | **212** | 1.41 | **5.62** | ACU(T) | 86 | 1.19 | 2.28 | AAU(N) | 63 | 1.08 | 1.67 | AGU(S) | 25 | 0.54 | 0.66 |
| AUC(I) | 88 | 0.59 | 2.33 | ACC(T) | 82 | 1.14 | 2.17 | AAC(N) | 54 | 0.92 | 1.43 | AGC(S) | 29 | 0.62 | 0.77 |
| AUA(M) | 119 | 1.31 | 3.15 | ACA(T) | 112 | 1.56 | 2.97 | AAA(K) | 64 | 1.47 | 1.70 | AGA(*) | 1 | 0.67 | 0.03 |
| AUG(M) | 62 | 0.69 | 1.64 | ACG(T) | 8 | 0.11 | 0.21 | AAG(K) | 23 | 0.53 | 0.61 | AGG(*) | 1 | 0.67 | 0.03 |
| GUU(V) | 84 | 1.49 | 2.23 | GCU(A) | 78 | 0.99 | 2.07 | GAU(D) | 33 | 0.94 | 0.87 | GGU(G) | 47 | 0.84 | 1.25 |
| GUC(V) | 47 | 0.84 | 1.25 | GCC(A) | **145** | 1.85 | **3.84** | GAC(D) | 37 | 1.06 | 0.98 | GGC(G) | 61 | 1.09 | 1.62 |
| GUA(V) | 73 | 1.3 | 1.93 | GCA(A) | 73 | 0.93 | 1.93 | GAA(E) | 62 | 1.36 | 1.64 | GGA(G) | 48 | 0.86 | 1.27 |
| GUG(V) | 21 | 0.37 | 0.56 | GCG(A) | 18 | 0.23 | 0.48 | GAG(E) | 29 | 0.64 | 0.77 | GGG(G) | 67 | 1.2 | 1.78 |

**Table S5** Base composition for the 13 PCGs in the three *Nanorana* species’ mitogenomes.

| Species | A(%) | T(%) | C(%) | G(%) | A+T(%) | C+G(%) | AT-skew | GC-skew |
| --- | --- | --- | --- | --- | --- | --- | --- | --- |
| *Nanorana parkeri* | 25.73 | 30.56 | 28.83 | 14.86 | 56.30 | 43.70 | -0.086 | -0.320 |
| 1st | 28.54 | 27.82 | 29.33 | 14.29 | 56.37 | 43.63 | 0.013 | -0.345 |
| 2nd | 25.31 | 30.79 | 26.85 | 17.02 | 56.12 | 43.88 | -0.098 | -0.224 |
| 3th | 23.33 | 33.07 | 30.32 | 13.26 | 56.41 | 43.59 | -0.173 | -0.391 |
| *Nanorana ventripunctata* | 25.88 | 30.55 | 28.57 | 14.97 | 56.45 | 43.55 | -0.080 | -0.310 |
| 1st | 28.48 | 27.90 | 29.20 | 14.40 | 56.39 | 43.61 | 0.010 | -0.339 |
| 2nd | 25.34 | 30.45 | 26.95 | 17.24 | 55.80 | 44.20 | -0.092 | -0.220 |
| 3th | 23.83 | 33.31 | 29.55 | 13.29 | 57.15 | 42.85 | -0.166 | -0.379 |
| *Nanorana pleskei* | 25.75 | 31.45 | 27.61 | 15.17 | 57.22 | 42.78 | -0.100 | -0.291 |
| 1st | 26.44 | 30.23 | 28.59 | 14.72 | 56.69 | 43.31 | -0.067 | -0.320 |
| 2nd | 25.47 | 30.66 | 27.48 | 16.36 | 56.14 | 43.86 | -0.092 | -0.254 |
| 3th | 25.34 | 33.47 | 26.74 | 14.43 | 58.82 | 41.18 | -0.138 | -0.299 |
